# Usage Patterns of GlucoNote, a Self-Management Smartphone App, Based on ResearchKit for Patients With Type 2 Diabetes and Prediabetes

**DOI:** 10.2196/13204

**Published:** 2019-04-24

**Authors:** Satoko Yamaguchi, Kayo Waki, Yasuhito Nannya, Masaomi Nangaku, Takashi Kadowaki, Kazuhiko Ohe

**Affiliations:** 1 Department of Prevention of Diabetes and Lifestyle-Related Diseases Graduate School of Medicine, The University of Tokyo Tokyo Japan; 2 Department of Ubiquitous Health Informatics Graduate School of Medicine, The University of Tokyo Tokyo Japan; 3 Department of Diabetes and Metabolic Diseases Graduate School of Medicine, The University of Tokyo Tokyo Japan; 4 Department of Pathology and Tumor Biology Kyoto University Kyoto Japan; 5 Division of Nephrology and Endocrinology Graduate School of Medicine, The University of Tokyo Tokyo Japan; 6 Department of Biomedical Informatics Graduate School of Medicine, The University of Tokyo Tokyo Japan

**Keywords:** telemedicine, mHealth, self-management, diabetes mellitus

## Abstract

**Background:**

Preventing progression from prediabetes to diabetes—or slowing the progression of diabetes—is an urgent task worldwide. Previous studies have shown that mobile health (mHealth) may powerfully support self-management for patients with type 2 diabetes. Certainly, mHealth improves health care efficiency and gives patients convenient access to self-management of their own health. Many health care apps are available right now, and their use in clinical studies with large-scale real-life data is expected. However, the usage patterns of those apps—especially in the absence of intervention by medical professionals—remain unknown.

**Objective:**

We developed GlucoNote, an app that uses Apple’s ResearchKit to support self-management for patients with type 2 diabetes and prediabetes; the app does not require prescription or intervention by medical professionals. We evaluated its usage patterns via a remotely conducted study.

**Methods:**

iPhone users across Japan who have type 2 diabetes or prediabetes were free to download GlucoNote and to participate in the study after they provided consent electronically on the app. The 522 users who enrolled in the study within 1 year of its release were analyzed. We analyzed the retention rates of 357 participants who recorded at least 1 of 4 items—body weight, blood sugar, blood pressure, or dietary information. Characteristics of participants who used GlucoNote longer than 4 weeks (*robust users*) were compared with those of participants who did not (*nonrobust users*). The changes among robust users were evaluated.

**Results:**

The median observation and retention durations were 382 days (interquartile range [IQR] 275-423) and 8 days (IQR 1-63), respectively. The retention rates for 2 days and for 4, 8, and 12 weeks were 0.627 (95% CI 0.575-0.675), 0.353 (0.304-0.403), 0.272 (0.227-0.319), and 0.220 (0.179-0.265), respectively. Men were more likely to be robust users than women (*P*=.02). At week 0, robust users were more likely than nonrobust users to have a higher daily energy intake (median 1595 [IQR 1198-1788] kcal vs 1451 [IQR 769-1657] kcal; *P*=.04) and have higher daily step counts (median 6108 [IQR 3797-9227] vs 5171 [IQR 2885-7258]; *P*=.001). Among robust users, body weight decreased from weeks 0 to 4 (mean 71.3 [SD 14.1] kg to 70.8 [SD 13.9] kg; *P*=.002) by mean 0.6% (SD 1.6).

**Conclusions:**

GlucoNote offered a valuable opportunity to evaluate usage patterns of apps. Future challenges include improving low retention rates and evaluating their effects.

## Introduction

Preventing progression from prediabetes to diabetes and slowing the progression of diabetes is an urgent task. Self-management is key to preventing that progression because there are a number of modifiable risks associated with prediabetes and type 2 diabetes; these include obesity, physical inactivity, and an unhealthy diet. Interventions using mobile phone apps to support self-management have proven to be effective in improving glycemic controls for diabetes patients [1,2]; such interventions also increase physical activity and reduce weight for overweight patients [3,4]. We previously developed DialBetics, a smartphone-based self-management support system for patients with type 2 diabetes. It provides real-time advice about lifestyle modifications based on patients’ data measured at home and the physical activities and diet they recorded. The system lets the medical staff remotely monitor the data and alerts them when those data reach critical values so a physician could intervene if necessary. A 3-month randomized study of 54 patients with type 2 diabetes has demonstrated that glycemic control was improved in the DialBetics group, whereas it did not improve in the non-DialBetics control group [5]. Although DialBetics proved to be effective, the number of patients with access to it was limited because only those who physically visited the outpatient clinic, received face-to-face instructions, and gave written informed consent could participate. Patients with prediabetes who do not regularly visit medical facilities could not be reached. Moreover, the system required continuous monitoring by medical staff, which can be costly.

Currently, numerous apps that support self-management of diabetes or obesity are available to the public and do not necessarily require prescriptions. Although these apps can attract a large number of users and are thus potentially a powerful tool, very few of them have undergone scientific evaluation. Rivera et al reported that of the 393 apps commercially available for weight loss, only 3 (0.8%) underwent scientific evaluation and only 1 (0.3%) reported the involvement of health care experts in app development [6].

We developed a novel app for patients with diabetes and prediabetes, taking advantage of our experience in developing DialBetics. To make an app available to a large number of users and evaluate its usage patterns, we used ResearchKit by Apple, one of the frameworks to create apps for medical research; it was released in March 2015 [7]. ResearchKit offers customizable functionality commonly used for medical research and lets investigators recruit and enroll patients entirely remotely, providing users with questionnaires to determine eligibility, obtaining electronic informed consent, and collecting biometric data, including daily step counts [8,9]. Several studies have reported apps using ResearchKit and many more are expected. Chan et al detailed the Asthma Mobile Health Study, which recruited 7593 participants from across the United States and detected increased reporting of asthma symptoms in correlation with weather conditions [10]. Bot et al reported the mPower Study, with participation by 1087 patients with Parkinson disease (PD) and 5581 without it [11]. This study administered questionnaires and structured tasks related to PD, taking advantage of ResearchKit features to provide surveys and real-time active tasks such as tapping motor activities. Crouthamel et al reported the Patient Rheumatoid Arthritis Data from the Real World app and collected patient-reported data about rheumatoid arthritis, including assessment of wrist range movement, measured by the smartphone-embedded gyroscope and accelerometer [12]. These studies using ResearchKit benefitted from a large enrollment that overcame geographical barriers.

Here, we report on the findings from GlucoNote, a self-management support app for patients with type 2 diabetes and prediabetes that we developed using ResearchKit. GlucoNote lets users self-monitor the data that they measure at home, the diet information they enter manually, and the number of steps counted by their iPhones’ built-in pedometers, which are displayed as graphs. At the same time, these data are sent to the server, letting the investigators evaluate the app’s usage patterns.

## Methods

### Design of GlucoNote

**Figure 1:**
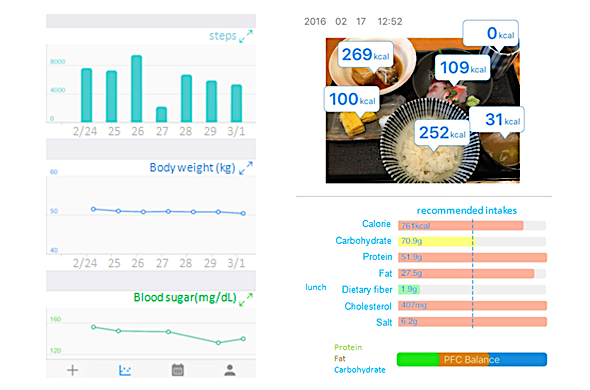
Sample view of GlucoNote screen.

The GlucoNote app was built with Apple’s ResearchKit [7]. Users enter body weight, blood sugar levels (fasting blood sugar or postprandial plasma glucose), and blood pressure levels. Changes in these parameters are displayed as graphs (Figure 1). Steps are counted by each iPhone’s built-in pedometer, and after the user sends the physical activity data to the server, step counts are displayed as a graph (Figure 1). Each time the user sends the physical activity data to the server, the step counts recorded in the past 30 days are also sent to the server, including the step counts of the 30 days recorded before study enrollment. If the user does not send the physical activity data, the step counts will not be sent to the server. GlucoNote facilitates easy input of dietary information by meal photos; this function was developed for DialBetics and is described in detail elsewhere [13]. To record dietary information, users can enter photos of a meal or choose from a menu list of 2913 items based on Eat Smart, a database provided by Eat Smart, Inc. When dietary information is entered from the menu list, the app automatically calculates each meal’s intake of calories, protein, fat, carbohydrate, dietary fiber, cholesterol, and salt, which are displayed as a graph. The recommended intake for 1 meal—calculated as one-third of the recommended daily intake—is also displayed for comparison (Figure 1). As it has been reported that self-monitoring is crucial in weight loss [14] and improved glycemic control [15], the GlucoNote app was designed to provide users with visual feedback of the parameters, which helps in self-monitoring of body weight, blood sugar level, physical activity represented by step counts, and diet. Physicians, including a diabetologist, nurses, and dietitians were involved in the development of GlucoNote.

### Eligibility Criteria for Participants

Before release, the study was approved by the Institutional Review Board of the University of Tokyo. All iPhone users aged ≥20 years in Japan with type 2 diabetes or prediabetes were eligible for the study after they provided electronic consent. They could download GlucoNote for free.

### Participants

**Figure 2:**
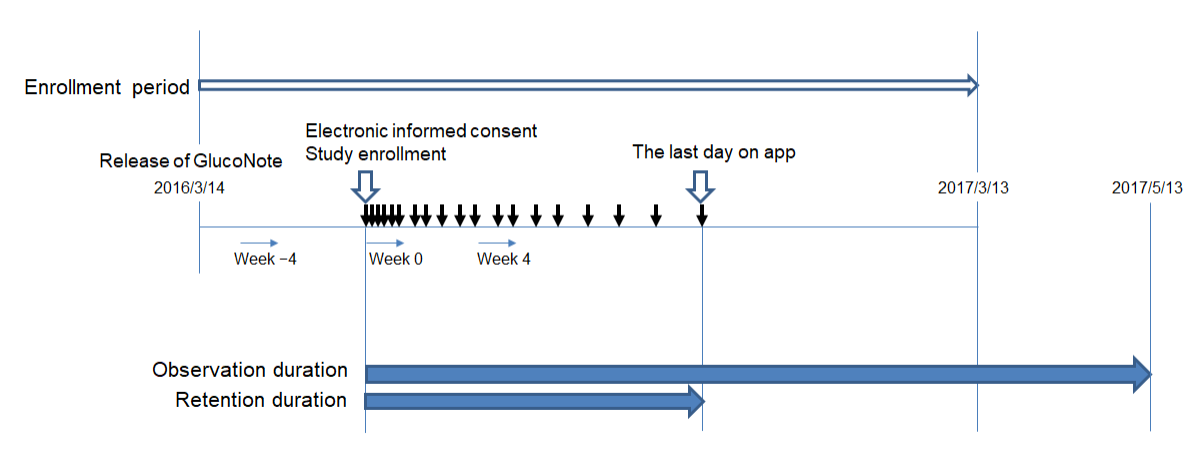
Definition of terms used in this study.

The GlucoNote app was made available on March 14, 2016, (but only in Japan) through the Apple App Store [16]. The GlucoNote release was announced in the homepage of the department and a press release was issued [17] (Multimedia Appendix 1). After downloading and opening GlucoNote, participants were questioned for eligibility, that is, “Are you 20 years old or older?”; “Have you been diagnosed as type 2 diabetes or prediabetes?”; “Are you able to understand and follow the consent forms?”; and “Are you living in Japan?”. Those who met the eligibility criteria proceeded to the informed consent screens. Before providing consent, participants had to read about the risks and benefits of participating in the study and their right to withdraw from it; they could withdraw at any time without giving reasons. Informed consent was by digital signature. Participants who enrolled between March 14, 2016, and March 13, 2017, were analyzed (Figure 2).

Participants were encouraged to fill in the following profile information: sex, body height, body weight, wake-up time, bedtime, smoking habits, age at diagnosis, presence of retinopathy, presence of neuropathy, and regular visit to a dentist. Target daily calorie intake could be calculated based on height and activity level.

Participants also filled in the results of medical examinations: date of examination, height, weight, waist circumference, blood pressure, blood sugar, hemoglobin A_1c_ (HbA_1c_), total cholesterol, high-density lipoprotein cholesterol, triglycerides, serum creatinine, aspartate transaminase, alanine transaminase, gamma-glutamyl transferase, urine protein, urine sugar, and urine albumin-creatinine ratio.

As shown in Figure 2, the observation duration was defined as the time from the day of study enrollment to May 13, 2017, 2 months after the end of enrollment. Retention duration was defined as the time from the day of study enrollment to the last day during the study period on which the user recorded at least 1 of 4 items: weight, blood sugar, blood pressure, or diet. Steps are different from other data—body weight, blood sugar, blood pressure, or dietary information—that require user input each time; they were automatically counted by the iPhones, and these data of steps recorded in the past 30 days were sent to the server at one time. Therefore, steps were discounted from analyses of retention rate.

### Data Analysis

The number of first-time app downloads was obtained from App Store Connect (Apple Inc), excluding app updates, downloads from the same Apple identification onto other devices, and redownloads [18].

For the participants’ demographics in Table 1, body mass index (BMI) was calculated from their first recorded body weight.

To compare the data between weeks, each participant’s mean values per week were calculated for weight, blood pressure, blood sugar, energy intake, or number of steps when the participant recorded those at least once during that week. When the user did not record that parameter for the entire week, the mean value of the corresponding week was treated as a missing value. When the user recorded the same parameter twice or more times on the same day, the data recorded first that day was used to calculate the mean value per week. The mean value recorded in the first 7 days starting from the day of study enrollment was defined as the value at week 0 (Figure 2). For the number of steps, the mean value recorded in the 7 days starting from 4 weeks before the day of enrollment was defined as the value at week −4 (Figure 2).

Daily energy intake was calculated by summing the calories of each meal when at least 3 meals were recorded per day. When only 1 or 2 meals were recorded, the daily energy intake for that day was treated as a missing value because one cannot tell whether participants did not record the meals or did not have the meals.

Retention rates were calculated by the Kaplan-Meier method [19], and the difference in retention rates between the 2 groups was evaluated by the log-rank test. The participants who continued to use the app to the end of the observation period were treated as censored cases and shown as vertical tick marks on the Kaplan-Meier curve. To compare characteristics between robust users and nonrobust users, the Fisher exact test was used for categorical variables and the Mann-Whitney *U* test was performed for continuous variables. A paired *t* test compared the data between week 0 and week 4 for parameters with a normal distribution, and a Wilcoxon signed-rank test was used for those with a non-normal distribution. Statistical analyses used R and Easy R [20].

**Table 1:** Demographics of participants in this study (N=522).

Characteristics		Statistics
**Sex, n (%)**		
	Men	417 (79.9)
	Women	101 (19.3)
	Unanswered	4 (0.8)
**Smoking status, n (%)**		
	Current smoker	32 (6.1)
	Ex-smoker	73 (14.0)
	Never smoked	95 (18.2)
	Unanswered	322 (61.7)
**Age at diagnosis (years), n (%)**		
	<40	45 (8.6)
	40-59	111 (21.3)
	≥60	10 (1.9)
	Do not know	12 (2.3)
	Unanswered	344 (65.9)
Hemoglobin A_1c_ (%), median (IQR^a^), (n=41)		6.3 (5.9-7.1)
**Height (cm), median (IQR)**		
	Total (n=489)	169 (164-174)
	Men (n=394)	171 (167-175)
	Women (n=91)	159 (156-163)
**Body weight (kg), median (IQR)**		
	Total (n=274)	70.6 (62.0-81.5)
	Men (n=222)	73.5 (63.8-82.2)
	Women (n=48)	61.2 (51.8-74.7)
**Body mass index** **(kg/m^**2**^), median (IQR)**		
	Total (n=270)	24.53 (21.99-27.82)
	Men (n=218)	24.62 (22.12-27.45)
	Women (n=48)	23.41 (20.36-28.95)

^a^IQR: interquartile range.

## Results

### Demographics of Participants

**Figure 3:**
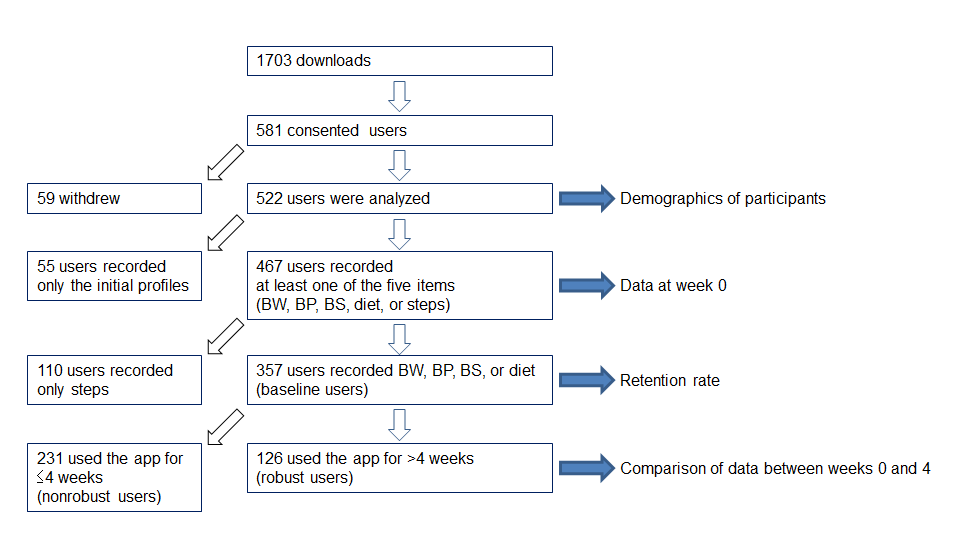
Study cohort description. BW: body weight, BP: blood pressure, BS: blood sugar.

After GlucoNote was released on March 14, 2016, it was downloaded 1703 times during the first year, with 581 users consenting to take part in the study. The daily number of downloads during the year is shown in Multimedia Appendix 1. Of these, 59 users withdrew and the remaining 522 were analyzed (Figure 3). Their demographics are shown in Table 1. Males accounted for 79.9% (417/522) of the users, their median body weight was 70.6 (interquartile range [IQR] 62.0-81.5) kg (n=274), and BMI was 24.53 (IQR 21.99-27.82) kg/m^2^ (n=270); 200 users answered the question about their smoking habit, 32 (16.0%; 32/200) of them being current smokers; 178 users answered the question about age at diagnosis, 111 (62.4%; 111/178) were diagnosed between the ages of 40 and 59 years; and 41 users recorded their HbA_1c_ level, the median value was 6.3% (IQR 5.9-7.1; Table 1).

**Figure 4:**
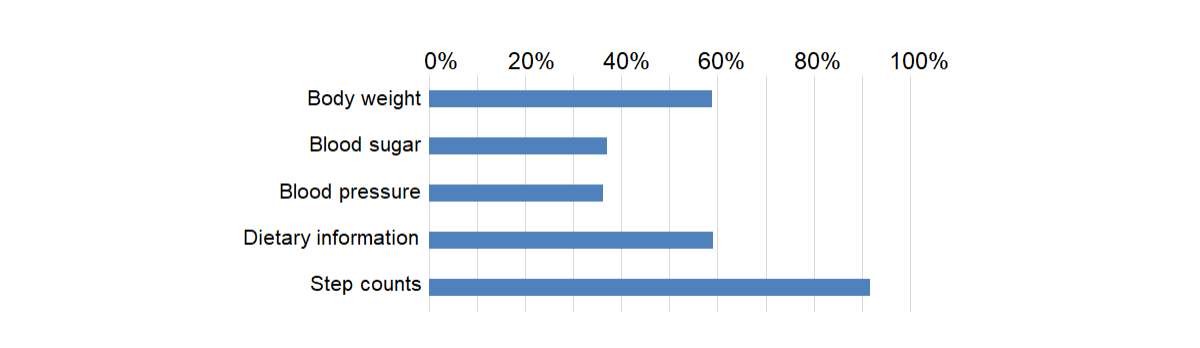
The proportion of users who recorded their data (n=467).

A total of 467 participants recorded at least 1 of 5 items: body weight, blood sugar, blood pressure, diet, or steps (Figure 3). The number of participants who recorded body weight, blood sugar, blood pressure, dietary information, or step counts was 274 (58.7%; 274/467), 172 (36.8%; 172/467), 169 (36.2%; 169/467), 275 (58.9%; 275/467), or 428 (91.6%; 428/467), respectively (Figure 4). The measured data of participants at week 0 are shown in Table 2.

**Table 2:** Recorded data of participants at week 0.

Variable	Participants, n	Median (interquartile range)
Body weight (kg)	244	70.3 (61.4-81.8)
Body mass index (kg/m^2^)	240	24.43 (21.91-27.83)
Fasting blood sugar (mg/dL)	124	118.5 (100.0-141.9)
Postprandial plasma glucose (mg/dL)	68	143.6 (121.8-177.9)
Systolic blood pressure (mmHg)	141	125.0 (116.8-133.0)
Diastolic blood pressure (mmHg)	141	79.4 (71.0-83.7)
Daily energy intake (kcal)	129	1510 (1003-1722)
Daily number of steps	409	5347 (3210-7716)

### Retention Rate

Retention rates were analyzed for 357 participants who recorded at least 1 of 4 items: weight, blood sugar, blood pressure, or diet (*baseline users*; Figure 3). With the median observation duration of 382 days (range 63-426 days, IQR 275-423 days), the median retention duration in *baseline users* was 8 days (IQR 1-63 days; Table 3).

**Figure 5:**
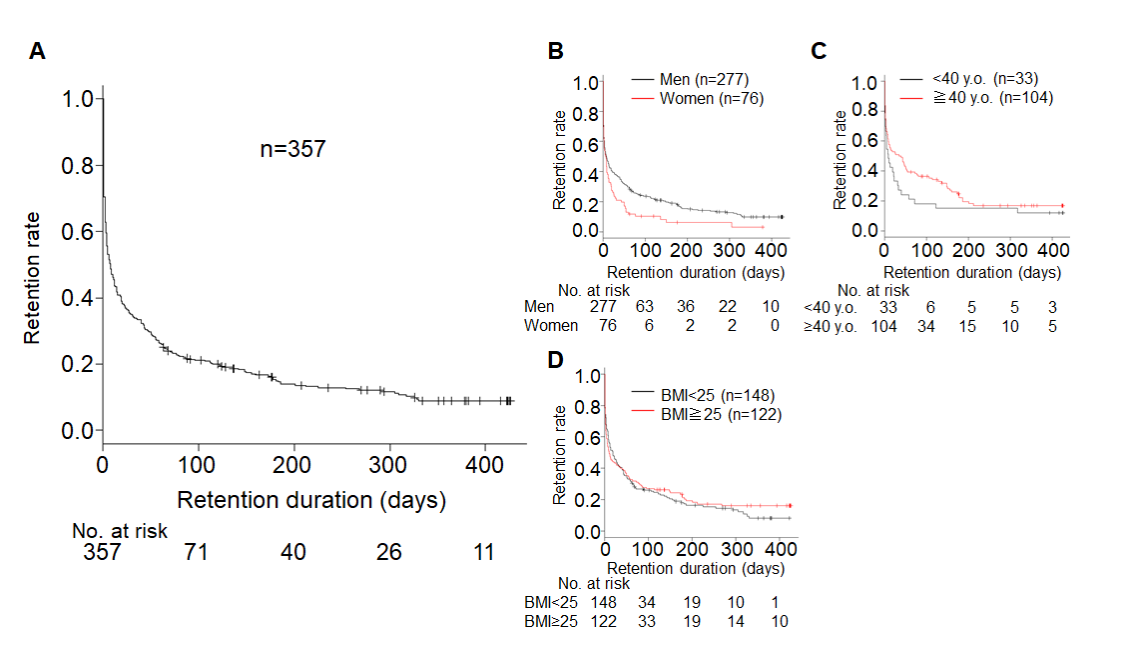
Retention rate of GlucoNote. Retention duration was defined as duration from the day of study enrollment to the last day on the app. (A) Retention rate of overall participants and according to (B) sex (men vs women, *P*=.01), (C) age at diagnosis (<40 vs ≥40 years old [y.o], *P*=.13), and (D) body mass index (BMI <25 vs ≥25kg/m^2^, *P*=.62).

Retention rates for 2 days and 4, 8, and 12 weeks were 0.627 (95% CI 0.575-0.675), 0.353 (0.304-0.403), 0.272 (0.227-0.319), and 0.220 (0.179-0.265), respectively (Figure 5). Male participants had a higher retention rate than female participants (*P*=.01; Figure 5). No significant differences were found between the groups with age at diagnosis <40 years and ≥40 years (*P*=.13) or the groups with BMI <25 kg/m^2^ and BMI ≥25 kg/m^2^ (*P*=.62; Figure 5).

Of the 357 participants, 126 used GlucoNote longer than 4 weeks (29 days or longer) and were analyzed as *robust users*. The median observation duration and retention duration for robust users were 375 (IQR 256-422) and 124 (IQR 60-275) days, respectively (Table 3).

Characteristics of robust users (n=126) and nonrobust users (n=231) were compared. Consistent with the result above, men were more likely to be robust users than women (*P*=.02); no difference was observed between the groups when age at diagnosis was <40 years and ≥40 years (*P*=.11; Table 3). Moreover, no differences in smoking status (*P*=.45), HbA_1c_ (*P*=.34), or body weight (*P*=.59) were observed. Those who answered the questions about smoking status and age at diagnosis were more likely to be robust users than were those who did not answer those questions (*P*<.001).

**Table 3:** Comparison of demographics of robust versus nonrobust users.

Variable		Total (baseline users; N=357)	Robust users (n=126)	Nonrobust users (n=231)	*P* value^a^
Observation duration (days), median (IQR^b^)		382 (275-423)	375 (256-422)	382 (279-423)	.38
Retention duration (days), median (IQR)		8 (1-63)	124 (60-275)	2 (1-7)	<.001
**Sex, n (%)**					.02
	Men	277 (77.6)	106 (84.1)	171 (74.0)	
	Women	76 (21.3)	18 (14.3)	58 (25.1)	
**Smoking status, n (%)**					.45
	Current smoker	23 (6.4)	9 (7.1)	14 (6.1)	
	Ex-smoker	64 (17.9)	35 (27.8)	29 (12.6)	
	Never smoked	80 (22.4)	42 (33.3)	38 (16.5)	
**Age at diagnosis (years), n (%)**					.11
	<40	33 (9.2)	11 (8.7)	22 (9.5)	
	≥40	104 (29.1)	53 (42.1)	51 (22.1)	
Hemoglobin A_1c_ (%), median (IQR), (n=40)		6.4 (5.9-7.2)	6.3 (6.0-6.8)^c^	6.7 (5.9-9.9)^d^	.34
Height (cm), median (IQR), (n=343)		169 (164-174)	170 (165-174)	169 (163-174)	.46
Body weight (kg), median (IQR), (n=274)		70.6 (62.0-81.5)	70.8 (62.0-79.9)	70.6 (62.0-82.5)	.59
Body mass index, median (IQR), (n=270)		24.53 (21.99-27.82)	24.49 (21.97-27.35)	24.57 (22.03-28.34)	.64
**Smoking status question, n (%)**					<.001
	Answered	167 (46.8)	86 (68.3)	81 (35.1)	
	Unanswered	190 (53.2)	40 (31.7)	150 (64.9)	
**Age at diagnosis question** **, n (%)**					<.001
	Answered	146 (40.9)	72 (57.1)	74 (32.0)	
	Unanswered	211 (59.1)	54 (42.9)	157 (68.0)	

^a^*P* value comparing robust users and nonrobust users using the Fisher exact test for categorical variables and Mann-Whitney *U* test for continuous variables.

^b^IQR: interquartile range.

^c^n=27.

^d^n=13.

**Table 4:** Comparison of measured data at week 0 between robust and nonrobust users.

Variable	Robust users (n=126)		Nonrobust users (n=231)		*P* value^a^
	n	median (IQR^b^)	n	median (IQR)	
Body weight (kg)	97	70.5 (60.0-79.4)	147	70.2 (61.8-83.0)	.26
Body mass index (kg/m^2^)	97	24.48 (21.72-27.02)	143	24.28 (22.00-28.44)	.38
Fasting blood sugar (mg/dL)	55	122.0 (106.0-140.9)	69	112.7 (93.3-144.5)	.08
Postprandial plasma glucose (mg/dL)	31	144.2 (136.0-184.1)	37	142.5 (118.0-160.0)	.24
Systolic blood pressure (mmHg)	68	125.3 (116.4-133.0)	73	125.0 (117.4-134.0)	.85
Diastolic blood pressure (mmHg)	68	79.8 (72.4-84.4)	73	78.0 (70.7-81.5)	.12
Daily energy intake (kcal)	70	1595 (1198-1788)	59	1451 (769-1657)	.04
Daily number of steps, week 0	119	6108 (3797-9227)	194	5171 (2885-7258)	.001
Daily number of steps, week −4^c^	107	5876 (3714-7975)	185	5594 (3308-7597)	.37

^a^*P* value comparing robust users and nonrobust users using the Mann-Whitney *U* test.

^b^IQR: interquartile range.

^c^For the number of steps, the values at week −4 were also compared between 2 groups.

Data at week 0 were compared between robust users and nonrobust users (Table 4). The daily number of steps at week 0 was higher among robust users than nonrobust users (median 6108 vs 5171; *P*=.001), whereas step counts at week −4 were comparable between the 2 groups (median 5876 vs 5594; *P*=.37). In addition, daily energy intake at week 0 was higher among robust users than nonrobust users (median 1595 kcal vs 1451 kcal; *P*=.04; Table 4). No significant differences in body weight, blood sugar, or blood pressure were observed.

### Comparison of Data Between Weeks 0 and 4

The data for robust users were compared between weeks 0 and 4 (Figure 2). Strikingly, body weight significantly decreased from week 0 to 4 (mean 71.3 [SD 14.1] kg to mean 70.8 [SD 13.9] kg; *P*=.002; Table 5). The mean difference in body weight between week 0 and week 4 was −0.5 kg (SD 1.2), corresponding to a mean decrease rate of 0.6% (SD 1.6). No changes in blood sugar level, blood pressure level, daily energy intake, or daily number of steps were observed between weeks 0 and 4 (Table 5).

**Table 5:** Comparison of robust users’ data between weeks 0 and 4.

Variable	Week 0	Week 4	*P* value^a^	Users, n
Body weight (kg), mean (SD)	71.3 (14.1)	70.8 (13.9)	.002	67
Body mass index (kg/m^2^), median (IQR^b^)	25.18 (21.96-27.64)	25.10 (21.76-27.10)	<.001	67
Fasting blood sugar (mg/dL), median (IQR)	122.0 (108.2-142.7)	123.0 (111.0-150.4)	>0.99	35
Postprandial plasma glucose (mg/dL), mean (SD)	171.3 (30.2)	145.8 (58.0)	.26	11
Systolic blood pressure (mmHg), median (IQR)	122.5 (113.6-131.4)	121.0 (112.9-127.9)	.053	47
Diastolic blood pressure (mmHg), mean (SD)	77.2 (8.0)	77.9 (8.0)	.55	47
Daily energy intake (kcal), median (IQR)	1595 (1273-1788)	1762 (1408-2012)	.06	46
Daily number of steps, median (IQR)	6745 (4141-9883)	6376 (3468-9080)	.11	104

^a^*P* value comparing data between week 0 and 4 using a paired *t* test or Wilcoxon signed-rank test.

^b^IQR: interquartile range.

After the observation period, a questionnaire was sent to the participants asking whether they found GlucoNote useful for health management. Only 22 out of 522 participants (4.2%; 22/522) replied: 4 (18%; 4/22) people found the app *very useful*, 6 (27%; 6/22) *useful*, and 12 (55%; 12/22) *not very useful*; none of the participants pronounced it *not at all useful*.

## Discussion

In this study, we evaluated a novel smartphone app, GlucoNote, which supports self-management in patients with type 2 diabetes and prediabetes and is established on the mobile health (mHealth) platform, ResearchKit. The study was conducted entirely remotely—including recruitment of participants and obtaining informed consent—without intervention needed by medical staff.

### User Demographics

In our study, nearly 80% (79.9%; 417/522) of the participants were men (Table 1). This is much more biased than expected, even taking into consideration that men, in Japan, are more likely to have diabetes or prediabetes than women (28.5% vs 21.4%, respectively) [21]. There have also been reports that men use information and communication technology (ICT) more frequently, have less ICT anxiety, and have a more positive attitude toward ICT self-efficacy than women [22-25]. Consistent with those reports, our recent survey of patients with chronic conditions suggested that male patients were more likely to express willingness to use a personal health record than female patients (paper in preparation). It has further been reported that women are more likely to perceive diabetes as a stigma than men [26], and it may be that women were reluctant to use an app that was plainly meant for diabetes patients. Efforts to remove such barriers for women may contribute to broadening usage. The bias was not because of the difference in ownership of smartphones: a 2018 survey in the Tokyo area reported that 77.9% of men and 80.9% of women aged between 15 and 69 years owned a smartphone [27].

To get enough participants, we had to forego gathering information such as age because the more the input required before setting up GlucoNote, the fewer comply—meaning fewer study participants. That is why complete user demographics are unavailable. Moreover, many values are missing from the users’ profiles, a problem shared by similar studies [10]. For example, only 38.3% (200/522) of the participants answered the question about smoking (Table 1). Not surprisingly, those who answered questions such as smoking status or age at diagnosis were likely to use GlucoNote longer (Table 3). Improvement in usability and security may be helpful to entice participants to enter more information. In our recent survey of patients with lifestyle-related diseases, *time and effort needed* and *concern over security* were identified as the main barriers to using such personal health records as health care apps (paper in preparation). For example, instead of relying on users to manually input the data, developing functions such as automatic recognition from a photo image of medical examination results may be useful—as may be reassuring users by improved security.

We do not have information about the use of other apps by GlucoNote users or people who visited the product page of GlucoNote, although that would have been helpful in identifying potential users.

### Retention Rate

A rapid decline in retention rates has been reported in other studies using ResearchKit [12,28]. Although the ResearchKit platform offers the advantage of completely remote recruitment and enrollment, lack of human communication may mean less motivation for participants to continue compared with studies conducted face to face. In this study, the 2-day retention rate was only 0.627 (95% CI 0.575-0.675; Figure 5), meaning that more than 1 in 3 participants used the app only for 1 or 2 days. The median retention duration was as short as 8 days (Table 3). However, long-term use is essential for the app to affect users. Clearly, additional efforts to improve retention rates are necessary. Our previous study using DialBetics—conducted at a university hospital—showed a much higher retention rate of over 70% at 3 months, partly because the research team nurse contacted the participants and encouraged them when they missed measurements for 2 weeks [5]. Such intervention by medical staff certainly helps improve retention, thought it obviously means a higher cost. Alternatively, some incentives built into the app—such as reward points for long-term users—may be helpful.

The characteristics associated with the retention time of mHealth have not been well studied. In a study of asthma patients who used ResearchKit, being female and older correlated with longer retention [10]. By contrast, in our study, men had a longer retention rate than women (Figure 5 and Table 3). Age at study enrollment was not collected, but age at diagnosis did not correlate with retention rate (Figure 5 and Table 3). As mHealth is relatively new, factors predicting willingness to use it—or good adherence to mHealth—have not been well understood. To deliver mHealth effectively, it is important to identify the characteristics that mark suitable candidates for the app.

Interestingly, robust users had significantly higher step counts at week 0 than nonrobust users (median 6108 vs 5171 steps; *P*=.001), whereas step counts were comparable before using the app (median 5876 vs 5594 steps; *P*=.37; Table 4), suggesting that using the app may have prompted an increase in step count by robust users but not by nonrobust users. In contrast to steps that were counted automatically, daily energy intake data may be incomplete because users may not have recorded all their food and drinks. The higher daily energy intake among robust users at week 0 (Table 4) might reflect a likelihood that these users input meal information more completely than nonrobust users, in turn, a reflection of robust user attitudes toward using the app in contrast with those of nonrobust users.

### Body Weight Decrease Among Robust Users

We observed a significant decrease in body weight (*P*=.002) after 4 weeks, with a mean decrease rate of 0.6% (SD 1.6; Table 5). Of 67 users with weight data for weeks 0 and 4, 47 (70%; 47/67) showed a decrease at week 4, suggesting that using the app might have prompted behavior modification leading to weight decrease.

It was previously reported that weight loss was the dominant predictor of reduced diabetes risk in patients with impaired glucose tolerance, with every kilogram of weight loss resulting in 16% reduction in risk [29]. A nationwide Japanese intervention program showed that for people with metabolic syndrome, a 1% to 3% weight loss after a 6-month lifestyle modification program resulted in a significant decrease in HbA_1c_ [30]. Although body weight loss in this study (mean −0.6% in 4 weeks) is only marginal and may be of little clinical significance, the long-term use of GlucoNote by improving the retention rate may lead to a reduced diabetes risk in patients with prediabetes. More importantly, it would be of interest to follow up to determine if the weight loss is maintained over time.

It is difficult to explain the exact reasons for weight loss only from the data recorded; no change in steps per day or energy intake was observed between weeks 0 and 4. However, one can speculate that some behavior changes undetectable by an app were prompted—such as increased physical activity other than walking. In addition, it is possible that the app failed to detect changes in diet because users may not have recorded all their food and drinks.

### Study Limitations

There are several limitations of this study. First, as discussed above, its major limitation is the low retention rate. Second, as noted above, the participants’ complete demographics were unavailable, and data collection was incomplete. Consequently, many analyses were based on different sample sizes, a common defect shared with similar studies [10-12]. Correcting this will require effort to improve response rates and collect more complete data. Moreover, only robust users were available for the analysis, increasing the risk of selecting bias. Third, the study design was free of controls and eligibility, including diagnosis of diabetes and prediabetes, and the measured data were entirely based on self-reports and had not been validated by medical professionals—a common problem shared by similar studies. Fourth, the overall number of downloads was lower than expected, with only 1703 in the first year after the release. A more effective way to inform people of the app is desirable. In addition, to widen its targets to include people with a metabolic syndrome, including overweight or even healthy people may be considered. Finally, to evaluate the effects of the app, a randomized controlled trial must be performed.

### Conclusions

We developed and released GlucoNote, a novel app that uses ResearchKit to support self-management in patients with type 2 diabetes and prediabetes. This afforded a valuable opportunity to evaluate usage patterns. Analyses of the participants who enrolled in the study within 1 year of the release revealed the potential advantages and challenges of GlucoNote. Future tasks include improving retention rates and evaluating its effects.

## Appendix

Multimedia Appendix 1 The number of daily downloads during the first year after GlucoNote release. The number of daily downloads (n=1703) and study enrollment (n=522) are shown.

## Abbreviations

BMI: body mass index
HbA _1c_: hemoglobin A_1c_
ICT: information and communication technology
IQR: interquartile range
mHealth: mobile health
PD: Parkinson disease
