# Supplementary figures and images for: Usage Patterns of GlucoNote, a Self-Management Smartphone App, Based on ResearchKit for Patients With Type 2 Diabetes and Prediabetes
Source: JMIR Mhealth Uhealth. 2019 Apr 24;7(4):e13204. doi: 10.2196/13204 (PMC6505564; doi:10.2196/13204)

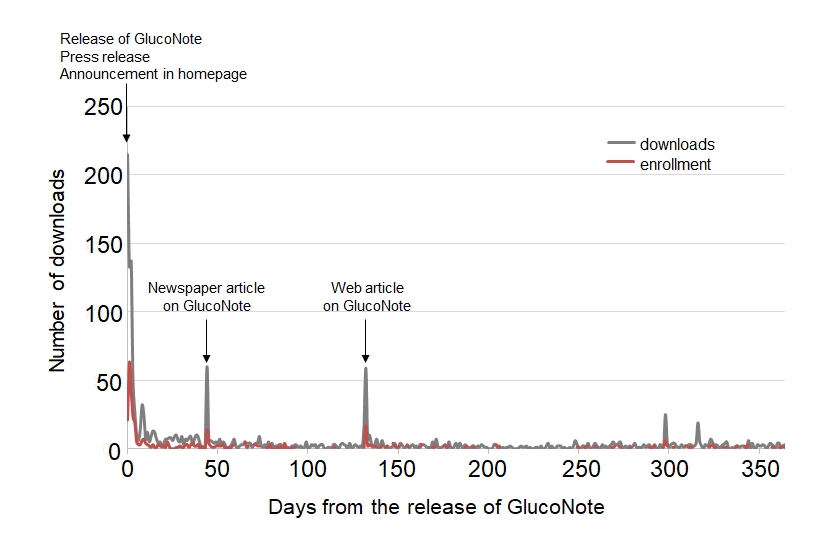

Supplement: Multimedia Appendix 1 [file mhealth_v7i4e13204_app1.png]
